# Cancer Genes: Origins and Directions

**DOI:** 10.3390/v18070702

**Published:** 2026-06-25

**Authors:** Peter K. Vogt

**Affiliations:** Department of Molecular and Cellular Biology, The Scripps Research Institute, 10550 North Torrey Pines Road, La Jolla, San Diego, CA 92037, USA; pkvogt@scripps.edu

**Keywords:** oncogenes, SRC, MYC, transduction, transcription

## Abstract

Avian viruses formed the foundation of early retrovirology. The historical line extends from the discovery of the first sarcoma virus by Peyton Rous to the quantitative determination of oncogenic activity in cell culture by the focus assay. As a viral group, avian retroviruses offered exclusive advantages that allowed the assembly of a unique and powerful tool chest for the analysis of viral activity. Among the fundamental discoveries facilitated by these tools were viral and cellular oncogenes, cell surface receptors, virus-specific detection of inapparent infection, high-frequency genetic recombination between retroviruses, and the genetic maps of simple retroviruses. The work with avian viruses was soon complemented by research on mammalian retroviruses, and several oncogenes that became the basis of successful targeted therapies were defined. The field of cancer genes is at a point of transition. Future developments will be driven by new technologies and interpretations. They will also require a more comprehensive approach.

## 1. Introduction

The purpose of this communication is to describe, analyze and assess the origins and consequences of cancer genes. The commentary consists of two parts, “Origins” and “Considerations”. The section “Origins” is not a comprehensive historical review or a coherent outline of discoveries, but a targeted and intentionally narrow selection of highlights that illuminate sources and growth in the field of cancer genes. The emphasis is on avian retroviruses, because this viral group provided unique technical advantages that made it the driving force of the field. The subject is restricted to genes that induce oncogenesis by gain of function; it does not include the broad category of tumor suppressor genes, which act by loss of function. The section “Considerations” is an attempt to point out developments that open new perspectives, but also to focus on deficiencies in our present understanding of cancer.

## 2. Origins

Research on avian tumor viruses defined the leading edge of early retrovirology. Two elements contributed to the prevalence of avian viruses at the onset of retrovirology: history and serendipity.

### 2.1. The Historical Origin

**Figure 1:**
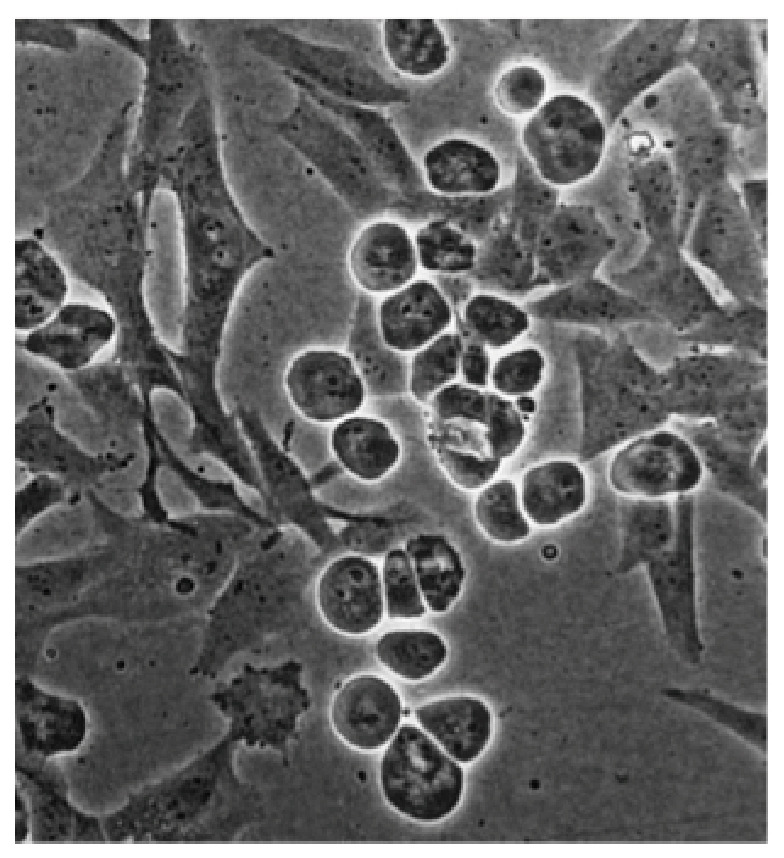
Chicken embryo fibroblasts infected by Rous sarcoma virus (RSV) form a focus of morphologically altered cells (adapted from [19]).

Tumor virology started with the discovery of a virus that could cause solid tumors in vertebrate animals. This discovery, made by Peyton Rous in 1910, marked the beginning of a scientific development that led to the recognition of cancer as a genetic disease [1]. In the first five decades after 1910, work with Rous sarcoma virus (RSV) was conducted exclusively in animals. It included studies on expanding the viral host range from chickens to other animal species and observations on the immune response to the virus [2,3,4,5]. By the middle of the last century, work with RSV had created a formidable body of knowledge, and innovative research was being pursued in several areas [6,7,8]. Then, in vitro cell culture, notably that of chicken embryo fibroblasts, became a widely adopted laboratory tool [9,10,11]. This technical innovation moved the attention in animal virology from animals to cultured cells. The critical impulse for that change came from phage research. The main representatives of that movement are the groups around Delbrück, Lwoff and Luria [12,13,14,15]. They followed strictly reductionist approaches and emphasized quantitation over description, ushering in the concept of quantitative biology. The principal technique of that scientific approach was the phage plaque assay [16]. Plaques are discrete areas of bacterial lysis induced by a single replicating viral particle. The phage plaque assay had been adapted to cytocidal animal viruses, with poliovirus and monkey kidney cells as first examples [17]. The approach was extended to Rous sarcoma virus, but instead of cell lysis, discrete foci of morphologically transformed cells in monolayers of avian fibroblasts became the indicator of viral infection and oncogenic activity [18]. This focus assay induced a fundamental change in the thinking of tumor virologists and became the backbone technique for early retrovirology. It is rapid, reliable and simple (Figure 1) [19]. In today’s cancer research, the focus assay is a fringe technique, but it has found new and valuable applications, thanks to novel vectors based on the RSV genome. It can now be used with a large number of cancer-inducing DNA sequences [20,21].

### 2.2. Serendipity

The serendipitous aspects contributing to the early prevalence of the avian field emerged from the unique structural and biological properties of avian retroviruses. These properties provided the components for building an exclusive, powerful, and novel set of tools and, thus, contributed decisively to our understanding of retroviruses.

The most important of these unusual properties of the avian retroviruses is the non-defectiveness of the RSV genome, combining the information needed for viral replication with the ability to transform cells [22]. RSV is unique in this respect. All other retroviruses carrying cellular oncogenes have exchanged viral genetic information for cellular sequences and, consequently, are defective in replication. But genomic non-defectiveness is a prerequisite for genetic analysis; and the groundbreaking studies of retroviral gene function and gene mapping depended on it. The singularity of non-defective RSV could reflect a unique feature of the viral capsid that allows the accommodation of a genome carrying an additional, nonviral gene.

Notations for RSV in the scientific literature always include a “strain” designation. The common strains are the Prague, Carr–Zilber, and Schmidt–Ruppin strains and the presumably independent viral isolate of B77. All of these are non-defective. The Bryan Hi-titer strain is an exception to this rule, carrying a defective genome that lacks the information for the viral glycoprotein. This defectiveness probably resulted from a method of virus production that selected for rapid growth and high yield. It is a strategy bound to generate short, defective viral genomes that depend on a ubiquitous helper virus that lacks an oncogene.

The second and less obvious of these serendipitous features consists of the multiplicity of viral surface glycoproteins. Avian retroviruses occur in nine distinct surface glycoproteins (Table 1) [23,24,25,26,27]. Each of these glycoproteins defines a viral subgroup and targets a specific cell surface receptor. Several of the most common cell surface receptors have been molecularly cloned. If they encode a dispensable gene, that gene can be deleted or inactivated, generating cells and animals that are genetically resistant to the corresponding subgroup of avian retroviruses (Table 2). More importantly, the viral glycoproteins give rise to the phenomenon of viral interference [28,29,30]. A cell infected with an avian retrovirus produces an excess of viral surface glycoprotein, which presumably saturates the cell surface receptor. Consequently, these cells become resistant to superinfection with a virus of the same subgroup but remain susceptible to infection with viruses of other subgroups (Table 3). Before DNA sequencing and nucleic acid hybridization became routine experimental techniques, viral interference was a specific and powerful tool to discover inapparent infection. Such inapparent infections are common with retroviruses, which can replicate in cultured cells without inducing any change in cell morphology or growth rate. However, their presence and subgroup affiliation can be readily detected by viral interference. Detection of inapparent infections is essential for genetic investigations. Viral glycoproteins and mutated cell surface receptors were also instrumental in the discoveries of high-frequency genetic recombination and of phenotypic mixing between retroviruses [31,32,33].

The findings of multiple glycoproteins, cell surface receptors, viral interference, and genetic non-defectiveness could be dismissed as merely archival information (Table 4), yet these tools played critical parts in experiments that led to fundamental discoveries. Here are two further examples:

A fast and reliable identification of viral surface proteins was essential in the experiments that showed early DNA synthesis in retroviral infection is virus-specific, which suggested the possible presence of a DNA polymerase in the viral particle [34]. It triggered the race that led to the discovery of reverse transcriptase [35,36].

The ability to detect inapparent infections led to the identification of the first oncogene, *src*. Serial passage of non-defective RSV commonly generates two types of progeny: one identical to the parental virus, capable of active replication and of inducing oncogenic cellular transformation, and a second one that produces infectious progeny without the ability to transform [30,37]. At the time, the transformation-defective virus was detectable only by viral interference, and this method revealed that, eventually, the transformation-defective virus prevails and constitutes most of the progeny virus. A possible reason for this enhanced fitness of the transformation-defective variant is a smaller genome that grows faster and, thus, replaces the parent. It has lost the genetic information that is essential for oncogenic transformation.

### 2.3. The src Paradigm

**Figure 2:**
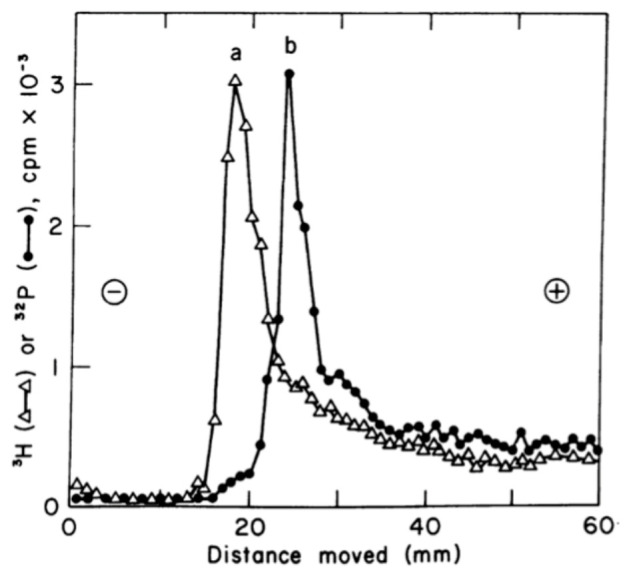
Viral genome size is correlated with oncogenic activity: The discovery of the src oncogene. Gel electrophoresis of RNA from an avian sarcoma virus capable of replication and oncogenic transformation (a). RNA from a transformation-defective but replication-competent derivative of the sarcoma virus (b) (adapted from [38]).

A comparison of genome length between transforming and transformation-defective viruses showed that this hypothesis is correct and that the RNA of the transforming virus is larger than that of the transformation-defective virus (Figure 2) [38]. This observation signaled the discovery of the viral oncogene *src*, the first principal proof of a molecular underpinning of the cancer gene hypothesis [38,39].

**Figure 3:**
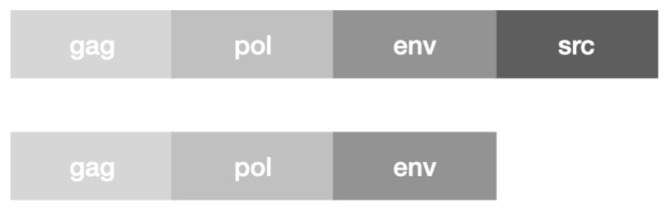
The src paradigm. Schematic representation of the genomes of an avian sarcoma virus capable of replication and of oncogenic transformation (**top**) and a deletion mutant of that virus that still replicates but cannot induce transformation (**bottom**). Gag, pol, and env are viral gene regions necessary for replication; src mediates oncogenicity.

Reverse transcriptase was discovered at the same time, and it opened the door to the molecular genetics of retroviruses [35,36]. Single-stranded DNA generated by reverse transcriptase from the genome of transformation-competent RSV was subjected to subtractive hybridization with the RNA of the transformation-defective variant. This allowed the purification of a single-stranded DNA probe specific for the *src* gene. The probe showed the presence of *src* sequences in the cellular genome, fundamentally changing the concept of oncogene from viral to cellular [40]. The corresponding SRC protein, discovered a few years later and shown to be a tyrosine kinase, provided a basic understanding of the regulatory role that oncoproteins play in cancer [41,42]. In the following years, a basic map of the RSV genome emerged that defined four protein-coding regions starting from the 5’ end: the viral *gag*, *pol*, and *env*, and the cell-derived *src* (Figure 3). Oligonucleotide mapping and conventional genetics, including temperature-sensitive mutants, genome deletions and truncations, were essential for achieving this result [43,44,45,46,47,48,49,50].

### 2.4. The Discovery of myc

**Figure 4:**
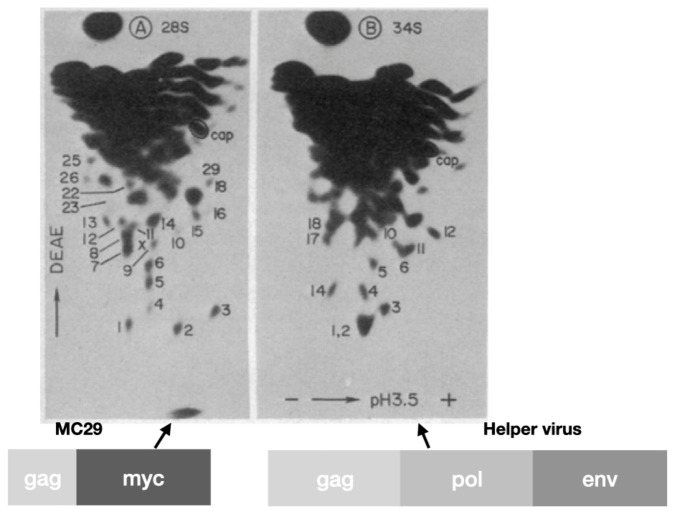
The discovery of *myc*. Oligonucleotide maps reveal unique sequences in MC29 (**left**, **A**) compared to the helper virus (**right**, **B**). The oncogenic virus MC29 is replication-defective, with a short genome that encodes a single gag-myc fusion protein (**left**, **A**). The helper virus genome (**right**, **B**) provides the missing replicating functions (adapted from [54,55]).

After *src* and RSV, there were other avian retroviruses that attracted attention. Among them was avian myelocytomatosis virus 29 (MC29), isolated in Bulgaria in 1967. Unlike common avian leukemia viruses, which induce inapparent infections, MC29 can form foci of transformed cells in avian embryo fibroblast cultures [51,52,53]. MC29 is replication-defective, and its genome is smaller than that of a replication-competent retrovirus. This size difference facilitated the separation of the MC29 RNA from that of its associated helper virus. Oligonucleotide fingerprints of MC29 RNA, and of the RNA of its helper virus, revealed non-viral sequences in the MC29 genome [54]. Complementary information came from the analysis of viral proteins produced by MC29-transformed cells. These cells synthesize a large protein, in which parts of the viral gag sequences are fused to non-viral sequences presumed to represent the oncogene (Figure 4) [54,55]. Together, oligonucleotide maps and protein analysis marked the discovery of a new oncogene, which was later termed *myc*. The non-viral sequences of MC29 were soon shown to be derived from the cellular genome, suggesting that the host cell is the universal source of viral oncogenes [56]. Following the genetic and structural analysis of the MC29 genome, the *myc* oncogene was also discovered in other avian retroviruses, including CMII, OK10, and MH2 [57]. The mechanism leading to multiple viral transductions of the same cellular oncogene is not understood. A possible explanation is provided by the retroviral life cycle. It includes recombination with the cellular genome that can lead to the acquisition of host sequences. With the exception of RSV, such acquisitions generate shorter, defective viral genomes that show enhanced fitness in replication.

The retroviral transduction of cellular genes with oncogenic potential provided the foundation for the genetic interpretation of cancer.

The MYC protein contains a dimerization and a DNA-binding domain, referred to as basic helix–loop–helix leucine zipper (bHLH-LZ) structure. The unliganded MYC is intrinsically disordered, but when it is bound to its partner protein MAX, forms a sequence-specific DNA-binding complex [58,59,60]. MYC was the first retroviral oncogene whose cellular counterpart was shown to be involved in human cancer [61]. It acts as a driving force in most human cancers [62,63,64,65], but has not been successfully targeted by a small, clinically approved inhibitor. However, the field of MYC inhibition is increasingly active [66,67], with interesting attempts to circumvent existing hurdles [68].

*MYC* was also the first cellular oncogene found to be activated by retroviral insertional mutagenesis [69]. It was shortly followed by the discovery of the *WNT* oncogene, activated by insertion of the mouse mammary tumor retrovirus genome into the host genome [70,71]. Activation of cellular oncogenes by retroviral insertion represents a distinct mechanism of tumorigenesis. It has been used to identify and characterize new genes and signaling pathways important in cancer [72,73].

Detailed biochemical analysis of the MC29-related retrovirus MH2 revealed that its genome contains, in addition to *myc*, a second, independently expressed, cell-derived insert termed *mil* [74,75]. At the same time, a cell-derived insert termed *raf* was found in the genome of a murine sarcoma virus [76]. *mil* and *raf* turned out to be derived from orthologous genes in the chicken and the mouse [60,77]. They represent a unique example of retroviral infection in two different classes of animals, leading to the transduction of the same gene. The Human Gene Nomenclature Committee has chosen the term *RAF* for the human ortholog. In the human genome, *RAF* represents a family of genes; one of these is referred to as *BRAF*. Mutated *BRAF* acts as an important driver in several human tumors, notably cancers of the colon and lung, and melanoma [78].

### 2.5. From Avian Erythroblastosis to HER2

Avian erythroblastosis is caused by a group of closely related retroviruses that are commonly designated as AEV “strains” [79,80,81,82,83,84]. Early molecular investigations used the AEV strain ES4 and identified two independently transcribed putative viral oncogenes, referred to as *erbA* and *erbB* [85,86,87], with *erbB* emerging as the likely driver of the disease. This possibility was confirmed by work on avian erythroblastosis virus strain H. Strain H carries only the *erbB* oncogene, yet it is fully capable of causing the disease [88,89]. *erbA*, a homolog of the thyroid hormone receptor, is connected to oncogenesis in a different context [90,91]. For *erbB*, the analysis of nucleotide and of amino acid sequences showed that it is a homolog of the epidermal growth factor receptor (EGFR) [92,93]. The EGFR family of proteins gained additional importance for cancer by the discovery of a related oncogene in a chemically induced tumor [94]. The gene, originally termed neu or erbB-2, but now referred to as HER2, also encodes an EGFR-related protein. HER2 is frequently amplified in breast cancer [95] and, like other EGFRs, it is expressed at the cell surface. That made it accessible to monoclonal antibodies leading to the development of the drug Herceptin [96]. Herceptin represents a prime example of successful targeted cancer therapy [97]. It is connected to avian erythroblastosis through a series of pivotal discoveries.

### 2.6. Finding New Viral Oncogenes

The incidence of cancer increases with age. Screening populations of old animals enhances the chances of making new discoveries. Chickens constitute the largest populations of animals that are professionally screened for tumors. Older birds, referred to as “retired layers”, used to be processed for human consumption and, hence, were inspected by a veterinarian of the Department of Agriculture. A single such processing plant would typically handle a minimum of 30,000 birds per day. Several spontaneous tumors detected in the course of two days in a plant in Los Angeles led to the discovery of multiple novel oncogenes. Three representative examples are *jun*, recovered from avian sarcoma virus 17 (ASV 17) [98]; *qin*, from ASV 31 [99]; and phosphoinositide 3-kinase (*pi3k*), from ASV 16 [100].

*Jun* was the first oncogene found to encode a transcriptional regulator. It is a leucine zipper protein and dimerizes with the related leucine zipper oncoprotein Fos to form the transcriptional activator complex AP1 [101,102,103].

The qin protein belongs to the family of Fox proteins and is now officially referred to as FoxG1 [99]. It is expressed exclusively in the telencephalon and is essential for the development of the brain in vertebrates. FoxG1 represents the only homeotic gene that has been recruited as an oncogene by a retrovirus.

Phosphoinositide 3-kinase (pi3k), a lipid kinase, became a promising drug target when overexpression and somatic mutation of the α isoform were discovered in human cancer [104]. The mutations are clustered in three sites of the gene and cause gain of function of the enzyme [104,105]. Expression of the mutated gene in cultured cells induces oncogenic transformation [106].

In the genomes of ASV17, ASV31, and ASV16, the 5’-ends of the respective oncogenes are fused to partial viral *gag* sequences, and in the virus-induced animal tumors, these oncogenes are expressed as gag fusion proteins. The gag sequence can contribute to the oncogenicity of the cellular sequences by determining cellular location or by enhancing the efficiency of translation.

In the following section of this commentary, we will briefly cite a few examples of oncogenes derived from mammalian retroviruses.

### 2.7. Ras

Two murine sarcoma viruses, the Harvey [107] and the Kirsten viruses [108], yielded oncogenic members of the Ras gene family, referred to as H*Ras* and K*Ras*. Early work was devoted to the genome structure of these viruses and the presence of host-derived sequences in their genomes [109,110,111]. The identification of the human H*RAS* as the determinant of the oncogenic phenotype of a human cancer cell line led to a burst of discoveries that showed the pervasive involvement of *RAS*, especially K*RAS*, in human cancer [112,113,114,115,116,117,118]. *RAS* is the most mutated oncogene in human cancer. The activating mutations map to a few hot spots of the gene and are single amino acid substitutions that lock the protein in its activated, GTP-bound form. Mutated RAS proteins are critical therapeutic targets. However, conventional paths to the design of small molecule inhibitors were ruled out by the apparent lack of suitable binding pockets and by the extremely high affinity of the protein for its target. These obstacles were overcome by fundamentally novel approaches that resulted in the identification of covalently binding, mutant-specific inhibitors [119].

### 2.8. Fos

Fos is the oncogene of the Finkel–Biskis–Jinkins (FBJ) mouse osteosarcoma virus [120,121]. In the virus-infected cell, it is expressed as a *gag*-*fos* fusion protein. The oncogenic activity of this protein is not dependent on its gag component, but on structural changes in the fos sequence [122,123], probably a result of the DNA-damaging agents that caused the original FBJ tumor. As already mentioned, fos binds to the jun protein using its leucine repeat sequence, forming the transcriptional enhancer factor AP1 [102,103,124].

### 2.9. Abl

The source of the retroviral oncogene *abl* is the Abelson leukemia virus [125]. A molecular investigation of its genome structure and expression detected a transformation-specific gag-abl fusion protein that functions as a tyrosine-specific protein kinase [126,127,128]. The significance of the *abl* oncogene for human cancer rests in the chromosomal translocation that fuses the human *ABL* gene on chromosome 9 to the *BCR* gene on chromosome 22, generating the fusion protein BCR-ABL [129,130,131]. BCR-ABL is a critical factor in the development of chronic myeloblastic leukemia [132,133]. The BCR-ABL fusion protein retains the kinase activity of ABL and was recognized as an attractive and promising drug target. Development of a small molecule inhibitor generated the compound STI571, marketed as Gleevec [134]. Therapeutic inhibition of BCR-ABL with Gleevec induces long-lasting remissions of chronic myelogenous leukemia [135,136]. Treatment with Gleevec or similar kinase inhibitors is now an important part in the standard of care of chronic myelogenous leukemia [136]. It is one of the most successful examples of targeted therapy.

## 3. Considerations

In this essay, we have followed an intentionally tight and confined path from the discovery of retroviral oncogenes to human cancer. It is a small segment of the abundant data that show that mutated genes play a critical role in the causation of cancer. Cancer genome landscapes reveal multiple somatic mutations that, in concert, contribute to uncontrolled cell growth [62,137]. Some of the most prevalent driver genes and their products were first identified in retroviruses. The functional significance of these oncoproteins is strongly supported by multiple dependency maps [138,139] (see also the cancer dependency project carried out at the Broad Institute, https://depmap.org/portal/ (accessed on 17 June 2026)). Cancer genetics is now fully integrated in clinical practice [140,141]. It is the origin and basis of targeted therapy. Cancer genetics is also the elementary tool for non-invasive early diagnosis [142].

However, advances in technologies, interpretations, and concepts are reshaping the field. Among the technologies that have undergone transformative developments are DNA and RNA sequencing and proteomics. Nucleic acid sequencing has made large gains in rapidity and can be scaled to single cell and single gene. New RNA sequencing methods have been introduced, and DNA sequencing has been expanded to long reads [143,144,145,146]. The results include novel insights into cancer genes and gene signatures, and the roles of genes in the origin and development of cancers [147,148,149]. New sequencing technologies have also led to a better understanding of the workings and failures of targeted therapy [150,151]. Additionally, they were instrumental in recognizing cellular plasticity as an important hallmark of cancer [152,153]. Plasticity, or mesenchymal drift, further plays a critical role in a recent proposal on the origin and nature of cancer, which integrates our knowledge of embryonal development and evolution in the process of oncogenesis [154]. Proteomics has undergone similar changes, with great improvements in speed and scale at single-cell level [155,156,157,158,159,160,161]. A technical fusion of proteomics and nucleic acid sequencing has also become an option [162].

Sequencing has further opened the door to an exploration of noncoding RNAs. The importance of the noncoding transcriptome as the genetic regulator of cell fate is experimentally supported and widely acknowledged [163]. One class of noncoding RNAs, the micro RNAs (miRNAs), has become an important part of cancer genetics, guiding diagnosis and treatment [164]. This discovery was facilitated by the fact that all miRNAs share basic molecular mechanisms of biogenesis and action [165]. But the large remainder of the noncoding genome is poorly understood. Most of these transcripts are long noncoding RNAs (lncRNAs, transcripts of more than 200 nucleotides). First discovered in a study of X-chromosome inactivation [166], they share several characteristic properties [167]. Although numerous publications have linked one or several lncRNAs to a specific cancer, there has not been a paradigmatic breakthrough that could integrate the entire class of lncRNAs into cancer development [167,168,169,170,171].

Ultimately, all questions that are raised by current cancer research merge into one unifying problem: the control of gene expression, specifically gene expression in higher vertebrates. The prevailing opinion of the control of transcription and gene regulation sees it primarily, if not exclusively, based on protein (transcription factor)–DNA interactions. The exclusivity of this view is almost certainly incorrect.

Early voices have pointed out the fundamental deficiencies and the inadequacy of these views, especially when applied to higher forms of life. One of them was Barbara McClintock, who focused on transposable elements as mediators of gene regulation [172,173,174,175].

A comprehensive hypothesis of the control of gene expression in the development and evolution of higher organisms was offered by Eric Davidson and collaborators [176,177]. They were the first to propose an essential role of regulatory RNA in the control of transcription. Their revolutionary concepts on hierarchical gene regulatory networks did not fit into the dominant views and, therefore, were initially ignored [178]. Today, the basic elements of the Davidson hypothesis are widely discussed [179,180,181,182,183], but have still not become part of the molecular biology canon.

A more recent voice emphasizing the centrality of regulatory RNA comes from John Mattick [184]. It is based on transcriptomic deep sequencing [185]. Mattick presents a compelling argument for the need of a “Kuhnian Revolution”, a paradigm shift, in molecular biology [186] and supports this with a comprehensive account of ideas and data from the past and the present in his book “RNA, the epicenter of genetic information” [163]. Contemporary reviews of transcriptional mechanisms and of RNA–protein interactions point in the same direction [187,188].

Much current work in biochemistry and chemistry is ultimately aimed at drug discovery. Proteins are the standard targets; RNAs are much less approachable. This situation creates a hidden bias that should not be extended to our understanding of the molecular mechanisms governing life. We need to remain open to ideas that contradict established dogma and that may have no immediate utilitarian value.

A final thought: Sequencing and proteomic technologies have introduced a fundamental change in the conduct of cancer research and of research in general. The reductionist fervor that started quantitative biology has yielded to a detached, statistics-guided analysis of large data sets. The philosophical consequences of this shift in approaching the unknown and in conducting science are frequently mentioned, but have not been fully explored [189].

## Figures and Tables

**Table 1:** Viral surface glycoproteins define avian retroviral subgroups.

Subgroup	Viral Glycoprotein
A	gp85-A
B	gp85-B
C	gp85-C
[…]	
J	gp85-J

**Table 2:** Viral surface glycoproteins interact with cell surface receptors.

Subgroup	Cell Surface Receptors
A	tv-a (dispensable gene)
B	tv-b (dispensable gene)
C	tv-c (dispensable gene)
[…]	
J	tv-j (dispensable gene)

**Table 3:** Viral interference.

Pre-Infecting Virus	Excluded Super-Infecting Virus					
Subgroup	A	B	C	D	E	J
A	★					
B		★				
C			★			
D				★		
E					★	
J						★

**Table 4:** Important features of avian retroviruses.

A non-defective sarcoma virus genome
The multiplicity of distinct surface glycoproteins
Specific interference by glycoprotein-receptor occupancy
Cellular resistance caused by receptor mutation.

## Data Availability

No new data were created or analyzed in this study.
